# 

**Published:** 1955-11

**Authors:** 


					THE
medical journal of the south-west
(Incorporating the Bristol Medico-Chirurgical Journal)
Established 1883
vol.
70 (vi)
November, 1955
NO. 258
PUBLISHED QUARTERLY
ANNUAL SUBSCRIPTION 16/- POST FREE
PUBLISHED UNDER THE AUSPICES OF THE
BRISTOL MEDICO-CHIRURGICALSOCIETY
Editorial Committee
Dr. J. E. Cates, Department of Medicine, Bristol Royal Infirmary. Editor.
Dr. A. H. Gale, The University, Bristol 8. Assistant Editor.
Dr. J. Macrae, Ham Green Hospital. Treasurer.
Dr. N. J. Brown, Southmead Hospital, Bristol.
Mr. J. P. Partridge, Southmead Hospital, Bristol.
Local Correspondents
Bath . . Mr. A. D. Bateman, 3 The Circus, Bath.
Exeter . . Dr. L. N. Jackson, Mount Jocelyn, Crediton, Devon.
Gloucester. Dr. R. F. Jarrett, 9 College Green, Gloucester.
Plymouth . Dr. C. R. Croft, Lexden, Hartley Av., Plymouth.
Taunton . Dr. M. McAlley, i The Crescent, Taunton.
Torquay . Dr. A. Robinson Thomas, 20 Devon Square, Newton Abbot, Devon*
Truro . . Dr. F. D. M. Hocking, St. Andrews, Perranporth, Cornwall.
Yeovil. . Mr. J. A. Vere Nicoll, Knapp House, Yeovil, Somerset.
NOTICE TO CONTRIBUTORS
Original articles are invited, provided they have not been published elsewhere, tj,ef
of forthcoming events, meetings held, degrees conferred, staff appointments, 0 ary
local news are welcomed. The editorial committee reserves the right to make H e
corrections. r
Illustrations should always be supplied ready for reproduction. All re-touching^,
other operations required will be charged to the author. Line drawings should be 0 js
in Indian ink. We regret that we must ask authors to pay for making blocks, u
necessary.
J. W. ARROWSMITH LTD., WINTERSTOKE ROAD,
BRISTOL.
Notes and News
Mr. Ashton Miller and Mr. J. H. Peacock have been appointed Hunterian Professors of ^e
Royal College of Surgeons, 1955-56.
Professor C. Bruce Perry has gone to the United States under the auspices of the Rockefel'e
Foundation to study methods of medical education. .jl
A series of Lecture-Demonstrations for general practitioners has been arranged and ^vl
commence on 16th October. j
The Long Fox Memorial Lecture will be delivered in the large Lecture Theatre at the
Fort at 8.15 p.m. on Tuesday, 25th October, by Professor C. W. Ottaway, PH.D., f.r.c.v.s., 0
"Art and Equine Anatomy". .
Mr. G. J. Hadfield has returned to the department of Surgery in the Bristol Royal Infirm3 :
after a year's study leave as a B.E.C.C. Travelling Fellow in the United States. His eX^'cc
on the growth of human breast cancer in animal hosts gained a first prize in the Scient'11
Section of the A.M.A. meeting this summer.
Appointments
UNITED BRISTOL HOSPITALS
Name Appointment Formerly
Mills, c.p., m.b., f.r.c.s. Senior Registrar, E.N.T. Registrar, E.N.T.
APPOINTMENTS 227
SOUTH-WESTERN REGIONAL HOSPITAL BOARD
July to September 1955
BRISTOL
Name Appointment Formerly
^Hillips, l. a., m.b., Anaesthetic Registrar, Southmead S.H.O. Anaesthetist, South-
b.s.(lond.). Hospital, Bristol. mead Hospital, Bristol.
^awson, l., m.r.c.s., Registrar in Chest Diseases, Medical Registrar, Papworth
L.r.c.p., m.b., ch.b. Bristol Chest Clinic and Hospital.
(Manchester). Associated Hospitals.
Freeman, J., m.r.c.s., Consultant E.N.T. Surgeon, Senior Registrar to the E.N.T.
L.r.c.p., m.b., b.s. United Bristol Hospitals (Joint Department, United Bristol
(lond.), f.r.c.s. (eng.), appointment with the South Hospitals.
d.l.o. Western R.H.B.).
Ganguli, P., m.b., Senior Registrar in Radio- Radiologist-in-Charge,
B.S. (calcutta), diagnosis, Southmead and Colonial Hospital, Port of
d.m.r.d. (liverpool), Frenchay Hospitals, Bristol. Spain, Trinidad.
D.M.R.T. (LIVERPOOL).
BATH
Name Appointment Formerly
Burrows, w. l. ., m.b., Re-appointed for further year as Is in general practice in
b.ch. (Belfast), m.d., Clinical Assistant in General Corsham.
M.r.c.p. Medicine, Royal United
Hospital (1 session).
Pinching, j., m.r.c.s., Re-appointed for further year as Is in general practice in
L.r.c.p., b.m., b.ch. Clinical Assistant in General Wells.
(oxon), m.r.c.p. (lond). Medicine, Royal United
Hospital (1 session).
Ellison, d. j., m.b., Appointed for one year as Clinical Is in general practice in
ch.b. (edin.), m.r.c.p. Assistant in General Medicine, Melksham.
(edin.). Royal United Hospital (1 session).
White, r. h. r., m.a., Medical Registrar, St. Martin's S.H.O. in Medicine, United
M.b., b.chir., m.r.c.s., Hospital and Royal National Bristol Hispotals.
L.r.c.p., d.c.h., m.r.c.p. Hospital for Rheumatic Diseases,
Bath.
Hodgson, j. h., m.b., B.s., Surgical Registrar, Bath Group of S.H.O., Wingfield-Morris
(sydney), primary Hospitals. Orthopaedic Hospital,
F.R.c.s. (eng.). Oxford.
Bishton, r. l., m.b., Consultant Pathologist to the Bath Lecturer in Pathology,
ch.b. (Birmingham), Clinical Area. University of Bristol.
M.d. (Birmingham).
SOUTH SOMERSET
Name Appointment Formerly
^Ligh, h. i., m.b., c.h.b. Surgical Registrar, Taunton and Registrar to the Department
(Bristol). Somerset Hospital, Taunton. of Neurological Surgery,
Frenchay Hospital.
DEVON AND CORNWALL
Name Appointment Formerly
P?Bins, r. h. c., m.a., Senior Orthopaedic Registrar, Registrar, Accident Service,
M.b., b.chir., f.r.c.s. Princess Elizabeth Orthopaedic Radcliffe Infirmary,
(eng.). Hospital, Exeter (from 1st Oxford.
January, 1956).
?Urke, n. f., m.b., b.ch. Orthopaedic Registrar, Mount S.H.O. in Casualty Depart-
(N.U.I.). Gold Orthopaedic Hospital, ment, South Devon and
Plymouth. East Cornwall Hospital,
Plymouth.
Parkes, J., m.r.c.s., Appointed for one year as Clinical Is in general practice in
L.r.c.p., m.a., m.b., Assistant in Paediatrics, Torbay Torquay.
E.chir., m.r.c.p. (lond.). Hospital and Rosehill Children's
Hospital, Torquay (2 sessions).
EiE>, a. a., m.b., b.s. Senior Psychiatric Registrar, Clinical Research appoint-
(durham), m.r.c.p. Moorhaven Hospital, Ivybridge. ment at St. Thomas's
(lond.), d.p.m. Hospital, London.
7 955 Index t0 Vol. 7o
Abercrombie, G. F., "General Practice",
120.
Adrenal Gland, Phaeochromocytoma of,
with Paroxysmal Hypertension, G. M.
Colson, 50
Andrews, C. T., Home Care of the Diabe-
tic, 193
Apley, John.?Medicine at Home and
Abroad, 16
Blindness, Observations on?R. Ramsay
Garden, 33
Bluestone, E. M.?The General and
Special in Medical Practice, 215
Bourns, Herbert K.?Casualty Depart-
ments, 168
Bryant D. and R. P. Warin.?Cat-Scratch
Disease, 113
Campbell, A. M. G.?Meningitis, 162
Casualty Departments.?Herbert K.
Bourns, 168
Casualty Department,What the G.P. Wants
from a, 167
Cat-Scratch Disease.?D. Bryant and
R. P. Warin, 113
Child Life in Past Times.?S. W. Hinds,
199
Clinico-Pathological Reports.?Mongolism,
Hypertrophic Pyloric Stenosis Treat-
ed with "Skopyl", and Death from
Miliary Tuberculosis and Meningitis,
97
Colloid Cyst of Third Ventricle, 127
' Jaundice with Hepatic Failure, 173
Haemorrhage from a Cavernous Haem-
angioma of the Cord Clinically Re-
sembling an Ascending Myelitis, 219
College of General Practitioners, South
Western Region Faculty, 61
Colloid Cyst of Third Ventricle?a
Clinico-Pathological Report, 127
Colonial Medical Research.?H. S. L.
Heller, 91
Colson, G. M.?Phaeochromocytoma of
Adrenal Gland with Paroxysmal Hy-
pertension, 50
Corner, Beryl D.?Meningitis in Child-
hood, 150
Cousin Marriage.?J. A. Fraser Robert?,
142
Craig, R. A.?The Changing Pattern of
Pulmonary Tuberculosis, 206
Creery, R. D. G.?Herpes Zoster Ence-
phalitis, 54
Damned Drugs, Part I.?G. E. F. Sutton,
7i
?Part II, 103
Diabetic, Home Care of the.?C. T.
Andrews, 193
Elderly Chronic Sick, The, 20
Foreword.?Sir Philip Morris, 1
Garden, R. Ramsay.?Observations on
Blindness, 23
"General Practice".?G. F. Abercrombie,
120
Haemorrhage from a Cavernous Haeman-
gioma of the Cord Clinically Resemb-
ling an Ascending Myelitis, a Clinico-
Pathological Report, 219
Heller, H. S. L.?Colonial Medical Re-
search, 91
Heron, A. Gordon.?That Backbone, 183
Herpes Zoster Encephalitis.?R. D. G.
Creery, 54
Herpes Zoster Generalisatus.?Mark Hew-
itt, 57
Hewitt, Mark, Herpes Zoster Generalisa-
tus, 57
Hinds, S. W.?Child Life in Past Times,
199
Jaundice with Hepatic Failure.?a Clinico-
Pathological Report, 173
Kitcat, C. de W.?Practical Points in the
Home Treatment of Tuberculosis, 79
"Marasmus".?A. V. Neale, 3
McRobert, Sir George R.?The Tropics
on our Doorstep, 83
Medical Practice, The General and Special
in. ?E. M. Bluestone, 215
INDEX
Medicine at Home and Abroad.?John
Apley, 16
Meningitis.?A. M. G. Campbell, 162
Meningitis in Childhood.?Beryl D. Cor-
ner, 150
Middlemiss, J. H.?A Radiologist Visits
Africa, 95
Mongolism, Hypertrophic Pyloric Stenosis
Treated with "Skopyl", and Death
from Miliary Tuberculosis and
Meningitis,?a Clinico-Pathological
Report, 97
Morris, Sir Philip.?Foreword, 1
Neale, A. V.?"Marasmus", 3
Nicoll, John A. Vere.?Diffuse Angio-
matosis of the Small Intestine Causing
Melaena, 47
Radiologist, A, Visits Africa.?J. H.
Middlemiss, 95
Radiotherapy, Some New Methods of.?
R. C. Tudway, 116
Roberts, J. A. Fraser.?Cousin Marriage,
142
Small Intestine, Diffuse Angiomatosis of
the, Causing Melaena.?John A. Vere
Nicoll, 47
Sutton, G. E. F.?Damned Drugs Part I,
7i
?Part II, 103
That Backbone.?A. Gordon Heron, 183
Tropics, The, on our Doorstep.?Sir
George R. McRobert, 83
Tuberculosis, Practical Points in the Home
Treatment of.?C. de W. Kitcat, 79
Tuberculosis, Pulmonary, The Changing
Pattern of.?R. A. Craig, 206
Tudway R. C.?Some New Methods of
Radiotherapy, 116
Underwater Breathing.?Ronald Woolmer,
9
Warin, R. P. and D. Bryant.?Cat-Scratch
Disease, 113
Woolmerj Ronald.?Underwater Breathing,
9

				

